# Different effects of spatial separation in action and perception

**DOI:** 10.3758/s13423-020-01867-9

**Published:** 2021-01-26

**Authors:** Sarah Schäfer, Christian Frings

**Affiliations:** Department of Cognitive Psychology, University of Trier, D-54286 Trier, Germany

**Keywords:** Action and perception, Common coding, Spatial distance, Cognitive categorization

## Abstract

Spatial distance of response keys has been shown to have an effect on nonspatial tasks in that performance improved if the spatial distance increased. Comparably, spatial distance of stimulus features has been shown to have a performance-improving effect in a (partly) spatial task. Here, we combined these two findings in the same task to test for the commonality of the effect of stimulus distance and the effect of response distance. Thus, we varied spatial distance in exactly the same fashion either between stimuli or between responses in a standard Eriksen flanker task. The results show that spatial distance only affected the processing of stimulus features, while it had no effect on the processing of response features. Regarding the idea of common coding of action and perception (Prinz, 1990), stimulus and response processing should be influenced by spatial distance in the same way so that our data might suggest a boundary for the idea of common coding.

Nowadays, there is consensus that action and perception are much more closely intertwined than it has been assumed in the 1980s to 2000s. In contrast to assumptions in those days, nowadays, theories claim a connection between perception and action in that the perception of a stimulus triggers potential responses automatically and thereby modulates behavior significantly (Frings et al., 2020; Hommel, 2004; Logan, 1988). Moreover, perception does not only influence action, but action and perception interact in a reciprocal manner. Thus, the simple intention to act also changes the perception of a stimulus (Gegenfurtner et al., 2010; Gegenfurtner et al., 2011). In the literature on action control, feature integration between different stimuli, between stimuli and responses, as well as between responses is analyzed comparably (Hommel, 1998, 2004; Moeller & Frings, 2019), suggesting a unification of aspects of perception and action. Additionally, feature integration and feature-based retrieval of previous episodes is functionally the same, independent of whether it is triggered by stimuli or responses (Frings et al., 2020). A further link between action and perception is highlighted by the fact that valence can be associated with a stimulus either via the emotional content of the stimulus or via an emotional response (Blask et al., 2016). In the same vein, in the realm of evaluative conditioning (Hughes et al., 2016; Walther et al., in press), valence can be learned via stimulus–stimulus, stimulus–response, or response–stimulus associations (Blask et al., 2016).

Carrying this commonality even further, the ideomotor theory postulates that even the anticipation of sensory effects of actions are used to guide behavior (for a review of the ideomotor theory, see Hommel & Wiers, 2017), and these (anticipated) sensory effects and motor programs share (at least partially) common neural representations—a principle known as common coding (Prinz, 1990; Rizzolatti, 2005). Thus, in several areas of cognition research, the originally postulated clear threshold between action and perception has dissolved. In that regard, many processes are assumed to work similarly on action and perception because it is assumed that the representation of action features and perceptual features is comparable.

## Coding of spatial distance of responses and stimuli

In the present study, we look at an effect that highlights this relation between perception and action. In particular, it has been argued that people use space—or, to be precise, the *perception* of space—to group and categorize items or objects (Cienki & Müller, 2008; Clark & Chalmers, 1998; Kirsh, 1995). One benefit of this function might be to save cognitive resources for other processes (Goldin-Meadow & Beilock, 2010). More specifically, Lakens et al. (2011) suggested that space could be used to separate nonspatial stimuli. In particular, they analyzed the influence of spatial response distance on the performance in binary (nonspatial) classification tasks. Here, participants classified the ink color of a letter string (i.e., either real color words or neutral letter strings, like *xxxx*) while ignoring its meaning. Participants responded faster if word meaning and color were congruent (e.g., if the word RED was presented in red ink) than if word meaning and color were incongruent (e.g., the word BLUE in red ink)—they observed the classic Stroop effect (Stroop, 1935; for a review, see MacLeod, 1991). More remarkable, the distance between the response keys (assigned to red and blue) was varied in such a fashion that in one condition the hands were close to each other and in the other condition hands were far apart. The authors observed smaller Stroop effects in the condition with far-apart hands as compared with the condition with close hands (Lakens et al., 2011). They interpreted this result as evidence for the influence of space on cognitive categorization. Specifically, it seemed to have been easier for the participants to mentally categorize the ink colors if the response keys that corresponded to the colors were far apart, as compared with a condition in which the response keys were close together.

Meanwhile, this finding has been replicated (Nett & Frings, 2014; Proctor & Chen, 2012, but here only partially) and has been interpreted in terms of the theory of event coding (Hommel et al., 2001). The idea is that the spatial features of the response (left vs. right) will be coded with the relevant features of the stimuli (i.e., the ink color blue vs. red). As a result, the discrimination between “blue-left” and “red-right” (for example) might be easier if left and right can be easily separated due to spatial separation. On a more general note, one can say that spatial separation of responses has been shown to influence the separation of visual stimuli (e.g., spatial response discriminability increased the salience of left–right coding; Koch et al., 2011; but see also Chen & Proctor, 2014 Jonas et al., 2014; Schiller et al., 2016).

If the commonality between the effect of spatial separation on stimuli and responses holds true, one might assume that spatial separation of stimulus features and response features is just the same—as action and perception are so closely intertwined (Prinz, 1990). Accordingly, it ultimately plays no role whether the separation is at the stimulus or the response level. Further, if response selection operates on stimulus–response bindings, the similarity of the codes included in these bindings determines the discriminability and hence response selection; anything that makes the units that selection has to select more different should speed up selection. If selection is based on event-files comprising stimulus and response features, it should thus not matter whether it is response or stimulus features that are being manipulated by spatial separation.

Undoubtedly, this is a very strong assumption, burdened by severe difficulties to test it. For instance, comparing spatial separation of particular (purely visual) stimulus features and particular response features touches on multisensory processing and integration (for a recent review, see Spence & Frings, 2020). Space is represented in different frames depending on input modality and very likely transferred to a visual-centered (or eye-centered) or at least eye-dominated reference frame. In other words, it is hard or even impossible to make a response (i.e., proprioceptive) distance and a visual distance exactly comparable. In addition, one can look at the distance between response keys or at the distance between the parts of the skin of the fingers pressing the keys (i.e., the somatotopic distance)—again, making it hard to judge whether spatial distance for response features should be modulated in external or somatotopic space. Considering these issues, we are going to put the assumption to the test to determine whether there is a commonality or a differentiation between the effects of spatial separation of stimuli and spatial separation of responses, and discuss the results against the backdrop of the mentioned issues concerning the representation of space.

## The present study

In the present study, we set out to investigate whether spatial separation affects the coding of stimuli and responses alike. Therefore, we used a flanker task (B. A. Eriksen & Eriksen, 1974; for a review, see C. W. Eriksen, 1995) because this task easily allows to manipulate spatial distance of stimuli and responses. Note that in many previous studies with the key-distance effect, the idea was to use nonspatial stimuli. As typical in the flanker task, participants had to classify a central target letter that was flanked by two adjacent distractors. These distractors could either interfere with responding to the target (incompatible trials) or they could facilitate responding to the target (compatible trials; see the Procedure section for further details). Importantly, we varied the spatial distance between these stimuli.

According to previous studies, flanker effects (i.e., the difference between compatible and incompatible trials) decrease with increasing spatial distance between the stimuli (e.g., B. A. Eriksen & Eriksen, 1974; Fox, 1998; Hommel, 1995; Miller, 1991). A first postulated decrease of the flanker effect due to a decreased retinal acuity at larger distances (Miller, 1991) has been extended to attentional explanations. Attentional factors like an attentional spotlight (LaBerge, 1983) might not exclude information from being processed (as flanker effects can occur even with larger distances), but may attenuate irrelevant stimuli (Hommel, 1995). Highlighting an influence of (perceptual) grouping on flanker effects (Baylis & Driver, 1992; Driver & Baylis, 1989; Fox, 1998), it becomes obvious that, when keeping other grouping factors constant, a larger distance between target and flanking stimuli results in a reduced interference effect. In addition to the variation of the distance between the stimuli, we varied the spatial distance between the response keys. If spatial distance facilitates the categorization of nonspatial stimuli and, additionally, if action and perception share common neural representations, then the flanker effect should diminish with increasing spatial distance between the responses as well. Considering several issues coinciding with this manipulation (as mentioned above), we mused that the one thing we can control and reliably measure is the *external* distance on the display and the *external* distance of the response keys. Importantly, we used spatial manipulations that previously yielded distance effects.

Intriguingly, and with the mentioned discussion of the common-coding approach in action and perception features in mind, we varied the spatial distance for stimuli and responses in three conditions and in exactly the same way (i.e., comparable distances between stimuli and responses), and we aligned the center of the stimuli with the center of the responses. Thus, we expected to see a drop in flanker effects from the close to the far distance (as reported by previous studies; e.g., Fox, 1998). Further, this decrease of the flanker effect should be similar for (external) stimulus distance and (external) response distance. Note that although we postulate a decrease of the flanker effect from the close to far distance condition, we do not make particular assumptions about the slope of the decreasing function (whether it be linear or quadratic, etc.). To foreshadow the results, we replicated the known effect of stimulus distance, but found no evidence for an effect of response distance.

## Method

### Participants

A total of 126 volunteers (87 females) with a median age of 22 years (ranging from 18 to 46 years) participated in the experiment. Participants gave informed consent before the experiment and received course credit for participation. All participants were naïve to the purpose of the study and reported normal or corrected-to-normal visual acuity. Two participants were outliers (i.e., far-out values according to Tukey, 1977) according to the number of errors they made (108 and 136 errors in comparison with a median number of errors = 25), indicating that they did not work through the task adequately. They were excluded from the analysis, resulting in a sample size of *N* = 124.

### Design

The experiment comprised a 2 (instance: *response keys* vs. *flanker stimuli*) × 3 (distance: *near* vs. *medium* vs. *far*) × 4 (target–flanker relation: *incompatible* vs. *compatible* vs. *identical* vs. *neutral*) within-participants design. Given the within-participants design and an *N* = 124, a power of 1 − β = .99 was given to detect even a small interaction effect (*f* = .10 as defined by Cohen, 1988; calculation were run with G*Power, Faul et al., 2007).

The distance was varied either for the response keys or for the flanker stimuli (the distance of the particular other instance was kept constant). The three distance conditions were orthogonally crossed with the target–flanker relation conditions for each instance. “Distance” as well as “instance” was manipulated block wise; the sequence of “distance” was randomized for each participant (and in each instance condition) and the sequence of “instance” (i.e., whether the distance between response keys or between target stimuli was manipulated first) was balanced between participants.

As the flanker effect is defined as both, an increase of response times (RTs) in trials with conflicting flanker stimuli (i.e., an interference in incompatible trials) as well as a decrease of RTs in trials with supporting flanker stimuli (i.e., a facilitation in compatible trials), we conducted the difference between compatible and incompatible trials as flanker effect. With regard to power considerations, we focused on the interaction in a 2 (instance) × 3 (distance) design, with the flanker effect as the dependent variable. Here, the sample size of *N* = 124 participants resulted in a power to detect even a small interaction effect (*f* = 0.1 as defined by Cohen, 1988) of 1 − β = .84 (α = 0.05; medium correlation of the repeated measures; calculations were done with G*Power 3.1.9.4; Erdfelder et al., 2009).

### Apparatus and material

**Fig. 1:**
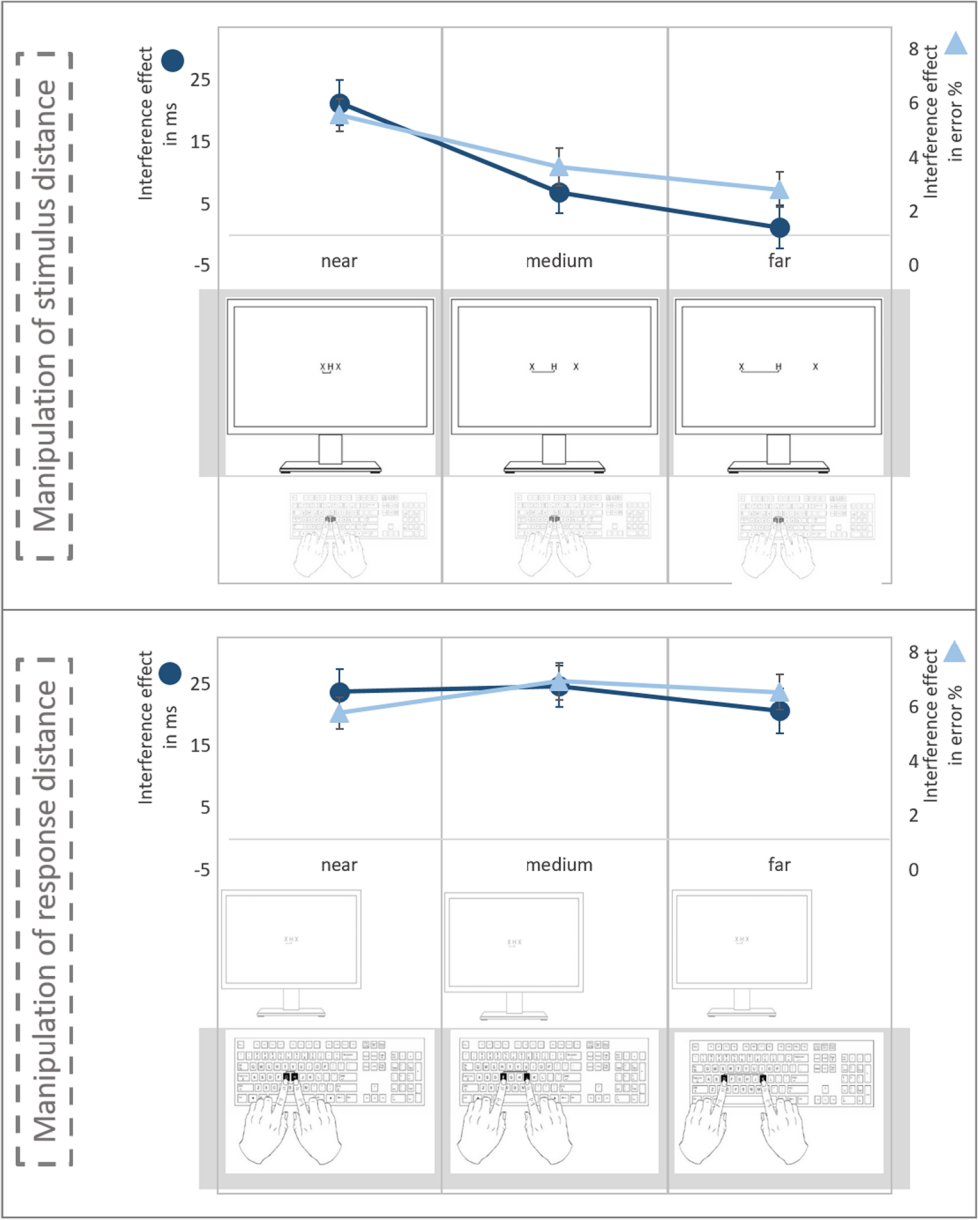
Graphical presentation of stimulus and response-key distance as well as the interference effect (depicted as the particular difference; see Results section) in RTs or error % in each distance-manipulation condition

Participants sat in front of a standard monitor with an unconstrained viewing distance of approximately 50 cm. Participants were instructed to respond to the letters *H* and *C* with the left index finger and to the letters *S* and *K* with the right index finger (all four letters could be either target or flanking stimulus). As a neutral flanking stimulus, the letter *X* was used. In the instance condition, in which distance was varied for the target stimuli, the *G* and *H* keys were to be used as response keys; in the instance condition, in which distance was varied for the response keys, the *G* and *H* keys were to be used for *near*, the *F* and *J* keys for *medium*, and the *D* and *K* keys for *far*. All visual stimuli appeared in white on a black background at the screen center. The letters were presented in Courier New bold font with a point size of 34. Hence, letters were presented with a visual angle of about 1.6° high. Target and flanking stimuli were presented with a distance of about 2.4° between each other (measured from stimulus center to stimulus center) in the response-keys condition. In the flanking-stimulus condition, they were presented with a distance of about 2.4° in the near-distance block, 7.6° in the medium-distance block, and 12.8° in the far-distance block (see Fig. 1 for a graphical presentation of the distance conditions). Note that the distances between the response keys were as best as possible comparable to the distances between the flanking stimuli: approximately 2 cm between the response keys and about 2.1 cm between the stimuli in the near condition, approximately 6 cm between the response keys and about 6.6 cm between the stimuli in the medium condition, and approximately 10 cm between the response keys and about 11.2 cm between the stimuli in the far condition. Furthermore, participants were instructed to place their hands on the keyboard in front of them (i.e., approximately 20 cm below and 30 cm in front of the display); hands were not covered.

### Procedure

At the beginning of the experiment, task instructions were given on the screen and summarized by the experimenter. The experiment consisted of a practice phase and an experimental phase. Following the instructions, 72 practice trials had to be worked through to practice the flanker task and the mapping of the target letters and response keys. Here, feedback was provided after each trial (either *correct* or *wrong* feedback in green or red font color, respectively) for 1,000 ms. Participants were instructed to respond as quickly and accurately as possible to the central stimulus as a target and to ignore the stimuli on the left and right side. After that, the experimental phase started with either the response-keys condition or the flanker-stimuli condition first (and the other instance condition following, sequence balanced). The response-keys condition started with an instruction about the possible response keys that would have to be used (colored markers indicated the near, medium, and far keys). Accordingly, the flanker-stimuli condition started with the instruction that the flanking stimuli would be presented with either a near, medium, or far distance (and that response keys would always be the *G* and *H* keys). Note that the effectors (i.e., the fingers) were the same throughout the whole experiment. Within one instance condition, each block of the distance conditions started with a slide that introduced the distance and told the participant to start the block with the space bar.

In each trial, one of the four letters (see Fig. 1) was presented as the target stimulus in the middle of the screen. Another letter was presented as flanking stimuli on the left and right side of the target. This letter could either be the same letter as the target (identical target–flanker relation), the neutral letter *X* (which was not mapped to any response and could thereby not evoke any response interference or facilitation; neutral target–flanker relation), or a letter that was mapped either to the same response as the target (compatible target–flanker relation) or to the particular other response (incompatible target–flanker relation). Note that two combinations should cause response facilitation (i.e., with the same and with the compatible flanker) and two combinations should cause interference (i.e., the two letters that are mapped on the particular other response) so that combinations with the neutral letter were presented twice as often as all other combinations in order to balance the proportion of compatible, incompatible, and neutral trials. All possible combinations resulted in 24 trials in each distance-condition block, and each trial was presented three times (resulting in 96 trials per block). Each participant worked through each distance condition (i.e., three conditions with 96 trials each) in both instance conditions, resulting in 576 trials in sum. The same proportions as in the experimental phase were realized in the practice phase. In both phases, trials were presented in random order. Each trial started with the presentation of a fixation cross for 500 ms, followed by the target–flanker combination, which was presented until a response was given. As soon as the response was given, a blank slide was presented for 500 ms, and then the next trials started.

## Results

Only correct responses with RTs above 200 ms and below three interquartile ranges above the third quartile of the individual RT distribution for each participant (far-out values according to Tukey, 1977) were used for the RT analysis. Averaged across participants, 92.7% of the trials were selected for RT analysis; 7.3% of the trials were excluded because of erroneous responses, and 2.1% were excluded due to the RT outlier criteria. Mean RTs and error rates for the overall design are shown in Table 1.

**Table 1:** Mean response times (RTs) in ms and error rates (ERs) in % as a function of instance (*response keys* vs. *flanker stimuli*), distance (*near* vs. *medium* vs. *far*), and target–flanker relation (*incompatible* vs. *compatible* vs. *identical* vs. *neutral*). Standard deviations in parentheses

			Instance					
			Response keys			Flanker stimuli		
			Near	Medium	Far	Near	Medium	Far
RTs	Target–flanker relation	Incomp.	548 (88)	537 (74)	540 (82)	535 (78)	516 (84)	503 (86)
		Comp.	524 (91)	512 (75)	519 (90)	513 (79)	509 (86)	501 (84)
		Ident.	520 (92)	518 (84)	521 (90)	513 (87)	507 (83)	503 (85)
		Neutr.	530 (87)	520 (76)	521 (81)	522 (84)	508 (81)	500 (80)
ERs	Target–flanker relation	Incomp.	8.4 (6.6)	9.7 (7.9)	8.8 (7.8)	8.5 (7.5)	6.8 (6.4)	5.9 (5.3)
		Comp.	2.7 (3.2)	2.7 (3.2)	2.3 (3.3)	2.9 (4.0)	3.1 (3.9)	3.1 (3.5)
		Ident.	2.5 (3.4)	2.6 (3.8)	2.9 (3.3)	2.6 (3.7)	3.4 (4.2)	3.1 (3.9)
		Neutr.	6.9 (7.5)	5.8 (5.8)	5.4 (6.3)	6.1 (5.7)	5.9 (6.2)	6.4 (6.0)

### Reaction times

A 2 (instance: *response keys* vs. *flanker stimuli*) × 3 (distance: *near* vs. *medium* vs. *far*) × 4 (target–flanker relation: *incompatible* vs. *compatible*, vs. *identical* vs. *neutral*) repeated-measures multivariate analysis of variance (MANOVA; see O’Brien & Kaiser, 1985, for the use of MANOVA analyzing repeated-measures designs), with flanker effects as the dependent variable, revealed a significant main effect of instance, *F*(1, 123) = 24.12, *p* < .001, η_p_^2^ = .16. This indicates that the flanker effect was larger when distance was varied for the response keys (and distance of stimuli was kept constant) as compared with when distance was varied for the stimuli (and, accordingly, distance of response keys was kept constant). The main effect of distance was also significant, *F*(2, 122) = 9.0, *p* < .001, η_p_^2^ = .13, suggesting that the flanker effect was significantly larger in the near condition than in the far condition. However, this effect significantly depended on the instance, which was indicated by a significant interaction of these two factors, *F*(2, 122) = 3.31, *p* = .040, η_p_^2^ = .05. An analysis of contrasts revealed that a linear trend—in detail a linear decrease—of the flanker effect with increasing distance was significantly influenced by the instance, *F*(1, 123) = 4.94, *p* = .028, η_p_^2^ = .04. This indicates that the linear decrease of the flanker effect due to increasing stimulus distance is different from the decrease of the flanker effect due to increasing distance of response keys (see Fig. 1).

### Error rates

We computed the same MANOVA on error rates (i.e., number of wrong responses per condition in %) and the results mirrored the data pattern in RTs. The main effect of instance was significant, *F*(1, 123) = 26.94, *p* < .001, η_p_^2^ = .18, indicating that the flanker effect was larger in the condition with varying response-key distance (compared with the overall flanker effect in the condition with varying stimulus distance). There was no significant main effect of distance, *F*(2, 122) = 1.36, *p* = .260, η_p_^2^ = .02, indicating that the flanker effect did not vary in dependence of the distance when collapsed about the two instance conditions. However, importantly, instance and distance interacted significantly, *F*(2, 122) = 4.92, *p* = .009, η_p_^2^ = .08, suggesting that the influence of distance was different for response keys and stimuli. Emphasizing the RT results, an analysis of contrasts with error rates also revealed that the linear decrease of the flanker effect with increasing distance was significantly different for responses and stimuli, *F*(1, 123) = 8.95, *p* = .003, η_p_^2^ = .07 (see Fig. 1).

## Discussion

We set out to investigate the effect of spatial distance between stimuli versus responses on interference effects (i.e., the flanker effect). Previous research suggested that (i) spatial distance of responses to nonspatial tasks improves performance, and (ii) feature representations in action and perception are assumed to share a common format. Based on that, we hypothesized that spatial distance should affect the coding of stimulus as well as response features in the same manner. Yet our results do not confirm this. Rather, while spatial distance of *stimulus* features led to the well-known decrease of flanker effects (replicating Fox, 1998), the exact same amount of spatial distance of *response* features had no impact on flanker effects.

The manipulation of the distance between the stimuli and the manipulation of the distance between the responses did not cause the same effects. However, regarding the difficulties about comparing spatial separation of particular stimulus features and of particular response features (which we mentioned in the Introduction), conclusions should be drawn carefully. It is important to note that, on the one hand, the literature about the key-distance effect revealed several modulating factors of the effect. For example, an importance of the conceptual distance rather than physical distance for the key-distance effect is assumed (Chen & Proctor, 2014). Phenomena like the near-by-hands phenomenon might play a role only in the response-distance condition as the phenomenon suggests that stimuli between the hands are processed differently than stimuli that are not placed between the hands (e.g., Abrams et al., 2008; Davoli & Brockmole, 2012). On the other hand, several aspects are quite apparent that might differentiate distance variation of a visual feature from distance manipulation of a response feature. For example, visual spatial resolution is far better than proprioceptive spatial resolution (e.g., for the finding that the localization of the position of the hand in space degrades quickly during visual occlusion, see Bowditch & Southard, 1882; Desmurget et al., 2000; for an influence of vision on proprioception and touch, see, e.g., Wesslein et al., 2014). Moreover, response coding has been assumed to be discrete and topological (see, e.g., the response-coding approach to the Simon effect according to Wallace, 1971). Given that participants saw their hands, it seems obvious that stimulus distance was purely visually processed, whereas response distance was processed at a multisensory level. Consequently, the influences of input from different sensory modalities and their interaction in the response-distance condition (for a review about multisensory processing and integration, see Spence & Frings, 2020) in many ways differentiates this condition from the purely visual stimulus-distance condition.

Nevertheless, we chose to manipulate the external distance because this is the one thing that we can control and reliably measure. Further, we chose approximately identical distances in the three distance conditions as well as aligned centers for the two instance conditions to make all conditions as comparable as possible. Additionally, the distances we used corresponded to distances that have been reported previously to produce effects—for stimulus distance in the flanker task (e.g., Fox, 1998) and for response distance in the Stroop task (e.g., Chen & Proctor, 2014; Lakens et al., 2011; Nett & Frings, 2014).

Remarkably, we found no hint of the previously reported key-distance effects (Lakens et al., 2011). The different possible modulating factors of the key-distance effect, which have been demonstrated to potentially eliminate this effect (Chen & Proctor, 2014; Proctor & Chen, 2012), cannot account for the null effect in our study (i.e., the effect is discussed to depend on conceptual distance between the response-distance conditions, physical distance between the hands, and additional keys between the two far response keys; all these factors were given in our study). Importantly, in most published studies on the key-distance effect, the task to measure cognitive categorization was the Stroop task, while we used a flanker task. Comparable to the nonspatial Stroop task, the target in the flanker task has no spatial feature that defines responding. Still, in the latter task, the target is selected via its location, thus making location a task-relevant feature. Even if the interpretation of the key-distance effect in terms of the theory of event coding (Hommel et al., 2001) would rather emphasize location as a task-relevant feature for the effect to occur, the current results might indicate that the influence of spatial distance of a response feature is different for spatial and nonspatial stimulus features and, remarkably, only affects performance for nonspatial stimulus features. In addition to the nonsignificant effect of spatial distance of a response feature, spatial distance of the stimulus feature did have an effect on performance (replicating Fox, 1998). Again, in the terms of the theory of event coding (Hommel et al., 2001), the coding of spatial features seems to depend on particular task demands (e.g., whether the task is per se spatial in nature). This assumption again points to distinguishable preconditions for the effects of space representations in action and perception, and thereby emphasizes our main interpretation that spatial coding of stimulus and response features is not completely interchangeable.

Taken together, we replicated the impact of spatial distance on the coding of stimulus features in the flanker task. Above that, under the exact same conditions, we did not replicate the previously reported key-distance effect in a flanker task. Consequently, as increased (external) spatial distance had significantly different impacts on the coding and processing of stimulus and response features, we conclude that a very strong position concerning common coding—namely, that coding of stimulus and response features is completely interchangeable—might be challenged. Even considering the difficulties when comparing space in action and perception, one conclusion might be that the impact of spatial distance is an important boundary for a simplified idea of the common-coding principle.
